# Familial Mediterranean fever concurrent with autoimmune glial fibrillary acidic protein astrocytopathy in a young adult

**DOI:** 10.1111/cns.14542

**Published:** 2023-11-23

**Authors:** Tingting Liu, Wu Yang, Xia Zheng

**Affiliations:** ^1^ Department of Critical Care Medicine, The First Affiliated Hospital Zhejiang University School of Medicine Zhejiang China

## INTRODUCTION

Familial Mediterranean fever (FMF) is a hereditary, autoinflammatory disease that presents with periodic fever and serositis.^1^, ^2^ To our knowledge, there is no report yet on FMF with concurrent autoimmune glial fibrillary acidic protein astrocytopathy (GFAP‐A), which is one of the common autoimmune astrocytopathies of the central nervous system (CNS).^3^ In this report, we describe the first case of FMF in a 24‐year‐old patient with co‐existing GFAP‐A. We also report for the first time a mutation (Chr16:3293424) at exon 10 of the MEFV gene via whole exome sequencing (WES), in the eastern Asian population. Although colchicine was not used, this patient's condition improved rapidly with immunoglobulin and steroid treatment.

## CASE REPORT

A 24‐year‐old man had high fever and headaches, sometimes accompanied by nausea and vomiting for 7 days. On June 21, 2022, he suddenly seemed to lose consciousness and was immediately admitted to the infection division. His neurological examination revealed merely unconsciousness and meningeal signs. Blood tests showed C‐reactive protein (CRP) level of 32.30 mg/L and serum creatinine (Cr) of 200 μmol/L. Brain CT results were normal. He neither had any chronic illnesses.

**FIGURE 1:**
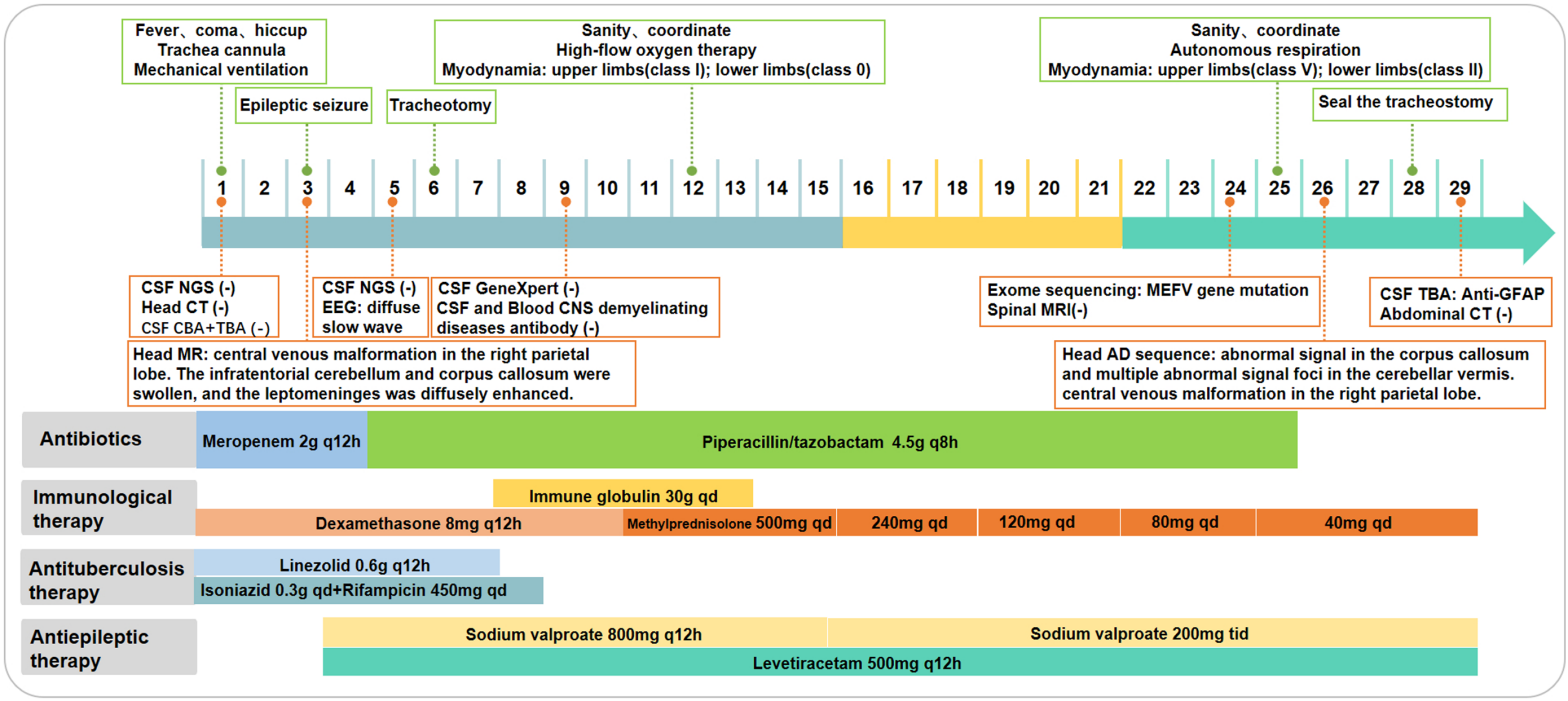
Summary of mechanical ventilation; antiepileptic, antibiotic, and anti‐tuberculosis drugs; and supportive treatment throughout the hospitalization course. CBA, Cell‐based assay; CSF, Cerebrospinal Fluid; EEG, Electroencephalogram; NGS, Next Generation Sequencing; TBA, Tissue‐based assay.

**FIGURE 2:**
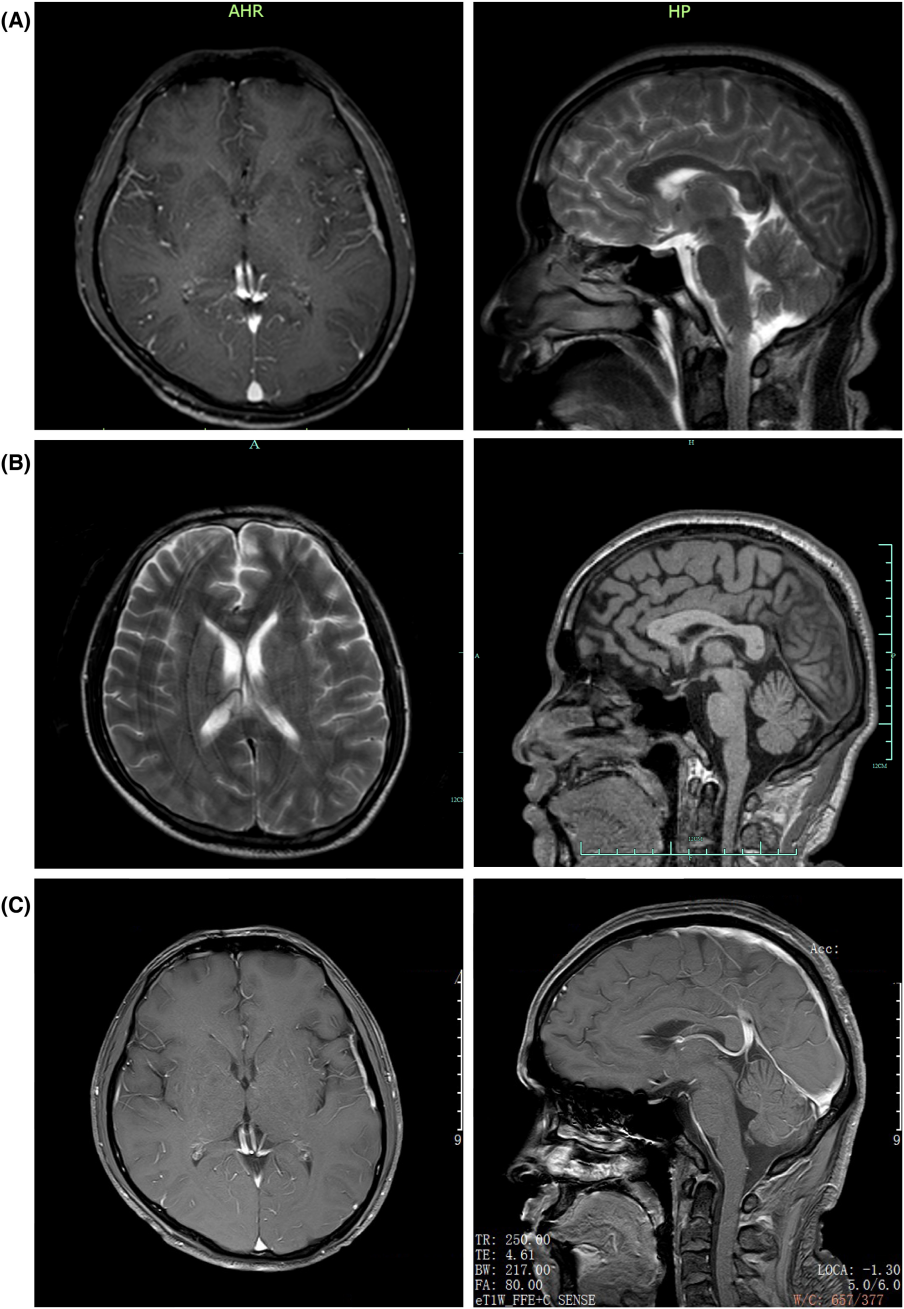
Brain MRI images of the patient. (A) Brain MRI on June 23, 2022. (B) Brain MRI on July 16, 2022. (C) Brain MRI on July 23, 2022.

The next day, the patient suffered from generalized seizures, accompanied by increased respiration rate and heartbeat, and oxygen desaturation; therefore, he was transferred to the intensive care unit (ICU). In the ICU, the patient was mechanically ventilated and treated with antiepileptic drugs (sodium valproate and levetiracetam), antibiotics (piperacillin/tazobactam and linezolid), anti‐tuberculosis medicines (isoniazid and rifampicin), and supportive treatment (Figure 1). Multiple CSF examinations showed pleocytosis (mainly lymphocytes), high protein level, and low glucose level. Other CSF examinations such as etiology inspection, self‐antigens, and myelin antigens showed normal results (Figure 1). Cranial magnetic resonance imaging (MRI) showed the infratentorial cerebellum and corpus callosum were swollen, and the leptomeninges were diffusely enhanced (Figure 2A). However, his electroencephalogram (EEG) showed diffuse slow waves. Based on his worsening condition, we used immunoglobulin and high‐dose methylprednisolone (Figure 1). Considering the rarity of the disease, trio‐whole exome sequencing (WES) and CSF/blood‐related autoimmunity antibodies were tested. The results of WES showed a heterozygous mutation of the MEFV gene, Cys688 located in exon 10 (Table 1). Detailed family history‐taking revealed that the patient's mother always has headaches, occasionally with abdominal pain. We genotyped the MEFV gene in his entire family, including his father, mother, and sister, and found the same MEFV gene mutation in his mother. We reexamined the cranial AD sequence and demonstrated an abnormal signal in the corpus callosum and multiple abnormal signal foci in the cerebellar vermis (Figure 2B). A repeat test revealed GFAP‐IgG in the CSF via tissue‐based indirect immune‐fluorescence test after several CSF autoimmune antibodies workup.

**TABLE 1:** Genetic analysis of *MEFV* depicting heterozygous mutations in exon 10.

Sample	Result	*MEFV*	chr16:3293424	c.2063A > G	p.Tyr688Cys
The proband	Heterozygous mutations	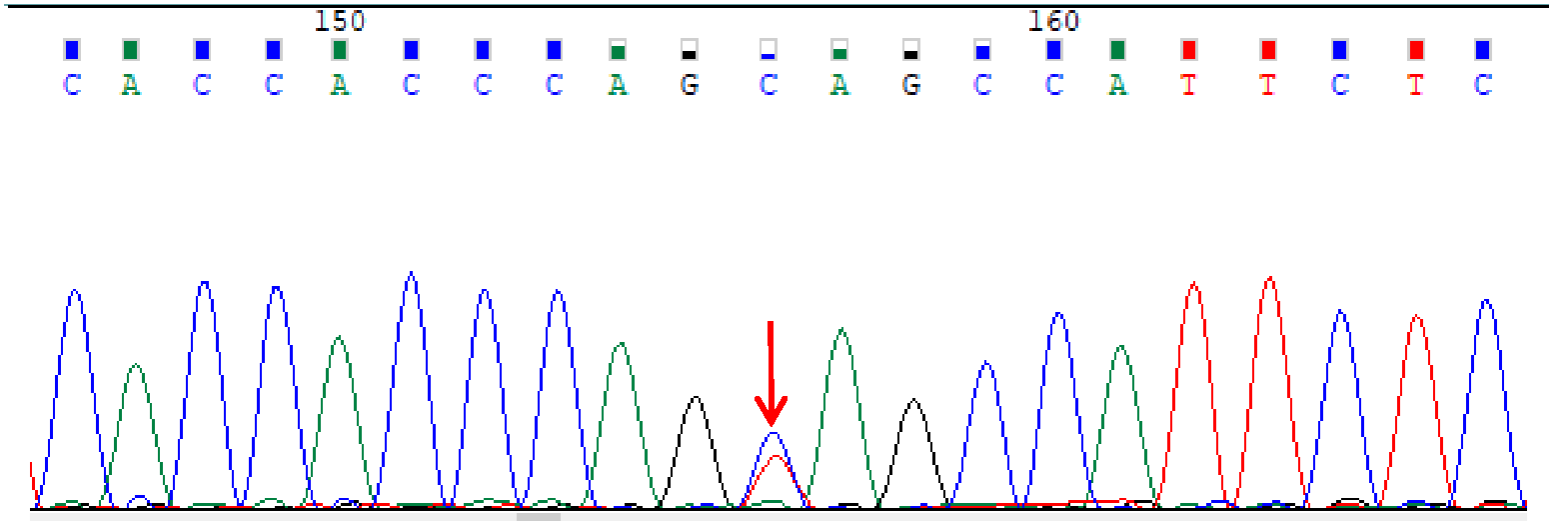			
Father of the proband	No mutations	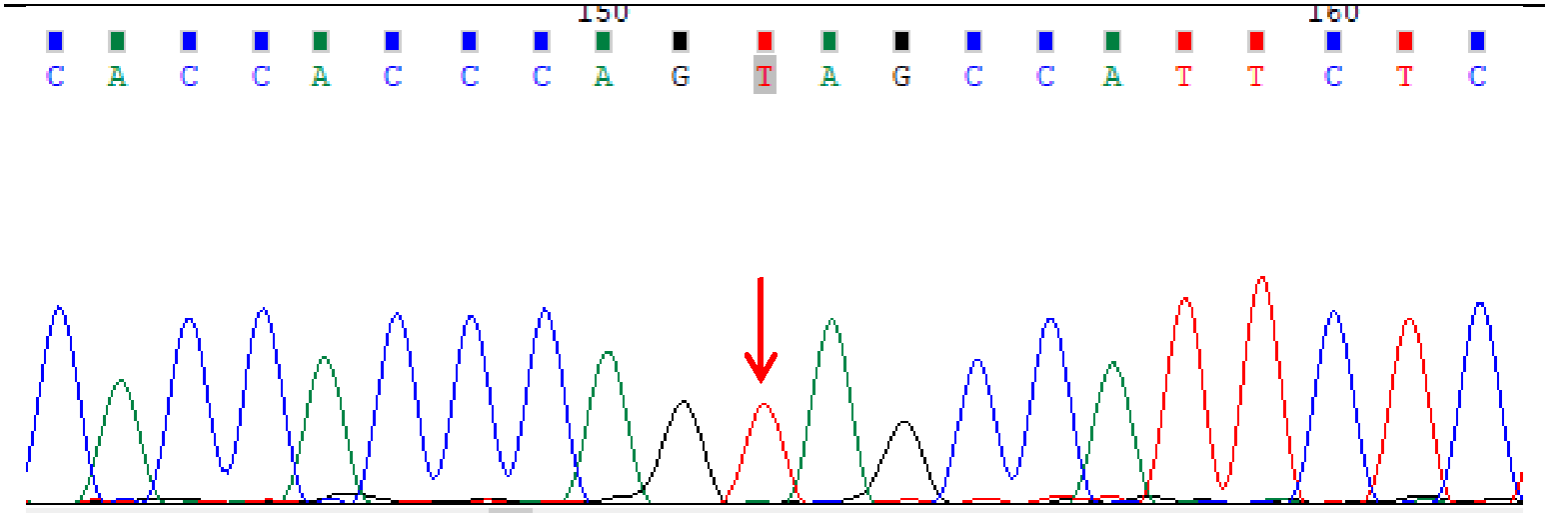			
Mother of the proband	Heterozygous mutations	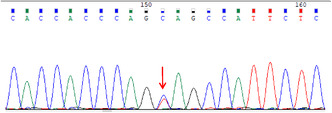			
Sister of the proband	No mutations	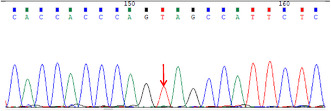			

After 2 weeks of treatment, the headache, fever, and seizure activity resolved. Neurological examination showed significantly improved results, such as consciousness, upper limbs power scale grade IV, and lower limbs power scale grade II. Cranial MRI showed no obvious lesions (Figure 2C). The patient then underwent rehabilitation to address the limb weakness, and the follow‐up is ongoing.

## DISCUSSION

FMF is one of the most common monogenic autoinflammatory diseases (AID) that mainly affects the ethnic groups originating from the Mediterranean basin.^4^ It is triggered by a mutation in the MEFV gene coding for pyrin, which is a key element of the inflammasome in the inflammatory process.^4^ FMF diagnosis depends on the presence of the MEFV mutation and typical clinical symptoms.^5^ The most common clinical symptoms of FMF are periodic fever, arthritis, and serositis. However, it is often asymptomatic and usually resolves spontaneously or flares suddenly.^5^

GFAP‐A is an autoimmune condition of the nervous system first defined in 2016.^3^ Patients with GFAP‐A present with symptoms of meningitis, encephalitis, and myelitis.^6^ The diagnosis of the disease is largely dependent on the detection and confirmation of GFAP autoantibody in the CSF.^6^ The recommended methods to detect this antibody include tissue‐based assay (TBA) and cell‐based assay (CBA). Other neurological findings are a high WBC count, high protein levels in the CSF, and brain MRI with characteristic linear perivascular radial gadolinium enhancement in the white matter perpendicular to the ventricle.^3^, ^7^ Multiple observational research has shown that steroid therapy could rapidly improve clinical conditions, and other treatments also include intravenous immunoglobulin (IVIG) and plasma exchange.^7^ Although a standard treatment regimen is still lacking, about 70% of patients respond well to steroid therapy.

To the best of our knowledge, this is the first reported adult case of FMF with concurrent GFAP‐A. The patient described herein suddenly developed untypical symptoms such as fever and headache, which were similar to meningoencephalitis. After several failed rounds of CSF/blood examination to identify the pathogenic factors, we prescribed anti‐tuberculous, anti‐viral, and anti‐bacterial medication. Despite treatment, the condition of the patient remained aggravated. We then used immunoglobulin and high‐dose methylprednisolone. At this time, WES and TBA methods (with CSF and blood samples) were used to detect the underlying cause of the disease. We separately detected a mutation) in the *MEFV* gene in the blood and GFAP‐IgG in the CSF. Of note, cys688 at exon 10 of the *MEFV* gene has never known the severity of FMF disease via database. However, the patient cooperated with the treatment and recovered well after treatment with immunoglobulin and steroids.

## AUTHOR CONTRIBUTIONS

Study concept and design: Tingting Liu, Wu Yang, Xia Zheng. Analysis and interpretation of data: Tingting Liu, Wu Yang. Drafting of the manuscript: Tingting Liu, Wu Yang. Revising it for intellectual content: Tingting Liu, Xia Zheng. Final approval of the completed manuscript: Wu Yang, Xia Zheng.

## CONFLICT OF INTEREST STATEMENT

The authors declare that they have no conflicts of interest.

## Data Availability

All data associated with this study are present in the paper.
